# Effects of Class II elastics on lower incisors during treatment with clear aligners vs. fixed appliance: a randomized clinical trial

**DOI:** 10.3389/fdmed.2025.1613037

**Published:** 2025-07-10

**Authors:** Giuseppina Laganà, Arianna Malara, Roberta Lione, Patrizio Bollero, Paola Cozza

**Affiliations:** ^1^ Department of Life, Health, and Health and Healthcare Professions, Link Campus University Rome, Rome, Italy; ^2^ Policlinico “Tor vergata”, Rome, Italy; ^3^ UniCamillus-Saint Camillus International University of Health Sciences, Rome, Italy; ^4^ Department of Systems Medicine, University of Rome “Tor Vergata”, Rome, Italy

**Keywords:** clear aligners, fixed appliance, lower incisor inclination, growing patients, IMPA

## Abstract

**Aim:**

To analyze the inclination of the lower incisor in patients with Class II malocclusion treated with elastics and clear aligners compared to a group wearing the same elastics and fixed appliance.

**Trial design:**

Prospective two-arms parallel group randomized clinical trial with a 1:1 allocation ratio.

**Materials and methods:**

A sample of 40 patients (19M and 21F), mean age 15.4 ± 1.8 years, was collected from the Department of Orthodontics at Policlinico Tor Vergata (Rome), and was randomly divided in two groups: Invisalign clear aligner group (AG), which consisted of 20 subjects (8M, 12F) and multibracket fixed appliance group (MBG), composed by 20 patients (9M, 11F). The subjects were selected according to inclusion criteria: full permanent dentition (excluding third molars), Class II molar relationship (2.5–4 mm), no history of orthodontic treatment. For each participant of the study, dental and aesthetic measurements, both millimeter and angular were performed on the lateral cephalogram at time T0 (before treatment) and time T1 (after 18 months of treatment).

**Results:**

In the short term (T1-T0 = 18 months), the analysis of the results showed no statistically significant changes in all evaluated parameters (IMPA, L1/A-Pg, Md1-TVL, LLA-TVL, *p* > 0.05). Therefore, there were no statistically significant change in the inclination of the lower incisors.

**Conclusions:**

The use of Class II elastics in AG group showed a better control of the lower incisors' inclination, compared to the MBG group. Therefore, aligners represent a good alternative in the correction of mild Class II malocclusion in cases where the lower incisors proclination is undesirable.

**Trial Registration:**

ClinicalTrials.gov (registration number: NCT06832475).

## Introduction

Class II malocclusion is the most common malocclusion (1, 2) and it is one of the main reasons for people to undergo orthodontic treatment. This alteration is characterized by an improper relationship between the maxillary and mandibular arches due to skeletal, dental or combined discrepancies (3). The treatment protocols may considerably vary depending on professional skills, malocclusion severity and patient compliance (4). Among all the well-known techniques, it is important to mention Class II elastics (5), used to camouflage mild skeletal Class II or to treat mild to moderate Class II occlusal relationship (6).

**Figure 1:**
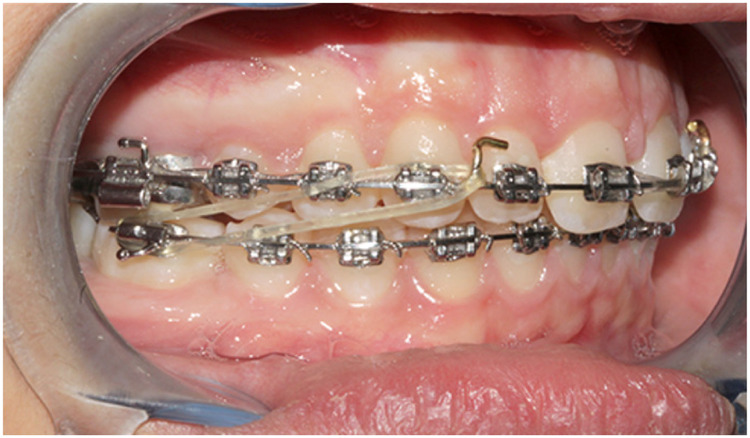
MBG sample with Class II elastics.

**Figure 2:**
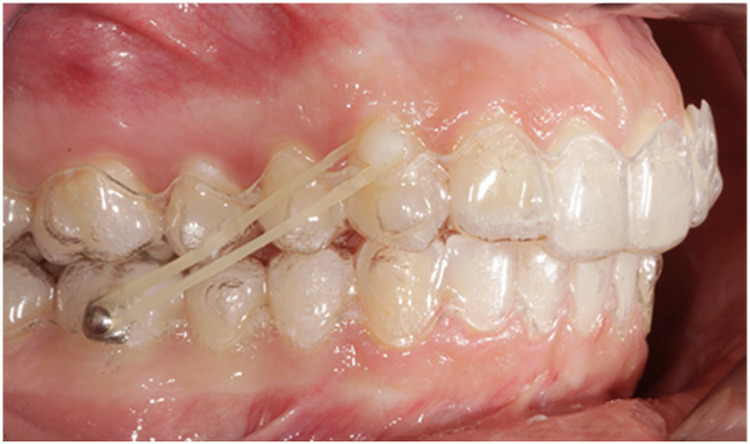
AG sample with Class II elastics.

**Figure 3:**
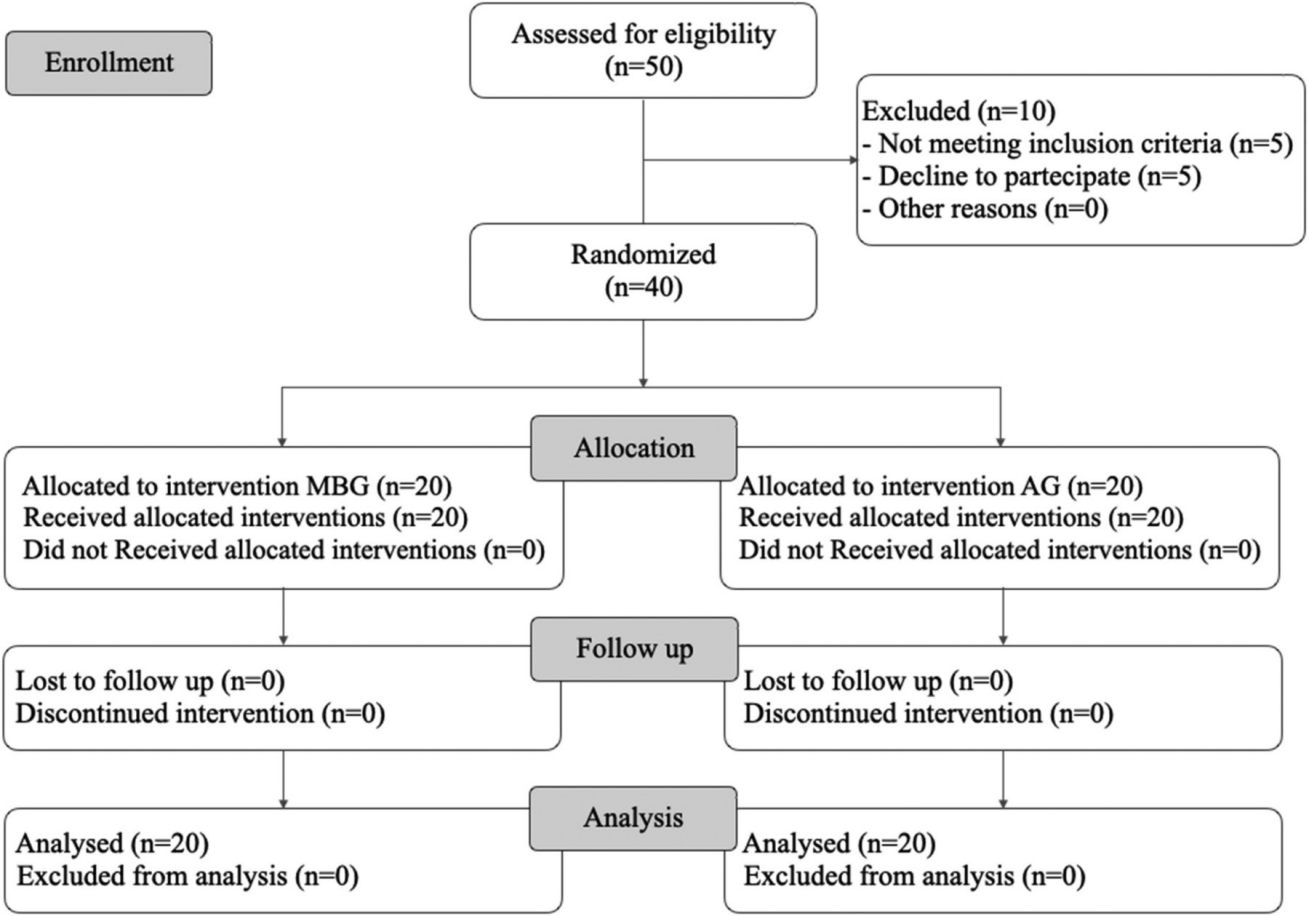
CONSORT flow diagram.

Intermaxillary Class II elastics are an effective method of clinical treatment in resolving aspects of malocclusion and have been part of the orthodontic armamentarium since first described by Maynard in 1843, then further in 1850 by Tucker, which still used gum elastics. Henry A. Baker was first to use latex elastics and to combine many of the concepts used by previous dentists into one orthodontic treatment modality referred to as “Baker anchorage”. The common use of elastics to correct Class II malocclusion has also been reported to cause reciprocal adverse effects (7). Side effects include both vertical and horizontal force vectors. This vertical force extrudes the maxillary incisors and mandibular molars and can lead to clockwise rotation of the occlusal plane. The horizontal vector of force has been shown to cause the mandibular first molars rotation or mesial tip, the flaring of lower incisors, the upper incisors retroclination and displace the entire lower dental arch anteriorly (6).

During the correction of Class II malocclusions by means of inter-arch elastics, special attention should be paid to the position of the lower incisors. Protrusion of the lower incisors by more than ±2 mm is unstable and high-risk condition because of gingival recessions, nevertheless, especially in non-extractive cases, it is often the first therapeutic choice (8, 9). Significant protrusion of the lower incisors can occur following the application of Class II elastics for a period more than three months, the application of removable functional appliances and the use of flexible arches, especially nickel-titanium ones, during the leveling phase of orthodontic treatment (10, 11). Nowadays, orthodontic treatment with removable clear aligners has become an increasingly popular alternative due to the growing number of adult patients who require esthetic and comfortable alternatives to traditional fixed braces (12–14).

However, in literature, few studies directly compared the effects of fixed treatment with the aligners one. The null hypothesis underlying this investigation is that there are no statistically significant differences in both studied groups.

Therefore, the purpose of this randomized clinical trial study was to analyze the inclination of lower incisors in adolescent patients with Class II malocclusion, treated with elastics and clear aligners compared to a group treated by elastics and fixed appliance.

## Materials and methods

### Study design

The Consolidated Standards of Reporting Trials (CONSORT) checklist was used as a guideline for conducting and reporting the present trial. This RCT was designed as a prospective two-arm parallel group randomized clinical trial with 1:1 allocation ratio.

This study followed the principles laid down by the World Medical Assembly in the Declaration of Helsinki 2008 on medical protocols and ethics and it was approved by the Ethical Committee of the University of Rome Tor Vergata (protocol number 34/23).

Written informed consent was obtained from all the parents of the subjects included in the study, after a clear and exhaustive explanation of the nature, purpose, and material risks of the proposed procedures.

The trial was registered on ClinicalTrials.gov (registration number: NCT06832475).

### Sample size

The sample size calculation was based on the main outcome: lower incisor proclination (IMPA).

The G*Power software version 3.1.9 (Universität Kiel) was used for sample size calculation.

Considering alpha = 0.05, beta = 0.20, *t*-test for paired data, and an effect size 0.7 (L1/GoMe mean difference = 4.3 grouped standard deviation = 5.8), at least 19 patients were required (15).

### Studied population

A sample of 40 patients, 19 males (M) and 21 females (F), mean age 15.4 ± 1.8 years was collected from the Department of Orthodontics at Policlinico Tor Vergata in Rome, from September 2022 to March 2023. The sample was randomly divided in two groups:

- **MBG** (multibracket group), consisted of 20 patients (9M, 11F) treated by straight-wire fixed appliance and Class II elastics
- **AG** (aligners group), consisted of 20 patients (12M, 8F) treated by Comprehensive Package Invisalign*®* system and Class II elastics.

### Inclusion and exclusion criteria

For both MBG and AG, the inclusion criteria were the following: full permanent dentition (excluding third molars), Class II molar relationship (2,5–4 mm), no or moderate crowding (0-3 mm), ANB angle between 4° and 6°, normo-divergence of the skeletal bases (FMA = 25° ± 3), no history of orthodontic treatment.

Exclusion criteria were not completed permanent dentition, skeletal Class II or dental Class II more than 4 mm, crowding more than 3 mm, subjects previously treated with orthodontic appliances.

Patients with dentofacial deformity or general medical problems, severe mandibular retrusion, poor compliance with aligners and elastics, multiple and/or advanced caries, impacted, missing, or supernumerary teeth, crowding more than 3 mm in lower arch, deep bite or open bite were excluded from the study.

### Treatment protocol

The treatment protocol for each group included non-extraction strategies, no IPR and the use of Class II elastics for at least 16 h per day. The elastic size was ¼″ and the force level was 6.5 oz. The elastics were used for a three-month period. The two groups used the same elastics in the standard position (upper canines—lower first molars).

For the MBG (Figure 1), the straight wire treatment included an aligning and leveling phase, achieved by using Nickel-Titanium arches (0.016/0.019 × 0.025 arch sequence) and a phase with heavier stainless steel rectangular arches (0.019 × 0.025) (16); during this last treatment phase patients were instructed to use Class II elastics for at least 16 h/day.

For the AG (Figure 2), the treatment provided the application of Invisalign clear aligner system and the absence of any other auxiliaries apart from Invisalign optimized attachments and buttons bonded on mandibular first molars for the application of Class II elastics.

Each subject was instructed to wear aligners for 22 h per day, except during meals and oral hygiene procedures and to replace aligners once a week, always the same day of the week. Patients were instructed to use Class II elastics for at least 16 h/day.

Every six the clinician personally checked the proper aligner fitting, the position of the attachments and the patient compliance. Patients wore Class II elastics for about three months.

Pre (T0) and post-treatment (T1) lateral cephalogram were collected from all selected patients. Treatment time was an average of 18 months (T1-T0 = 18 months).

### Randomization, allocation concealment and blinding

Allocation of patients to the two groups was determined by a computer-generated randomization list using Rv.0.1 software (26) and by a block size of 4 (Figure 3). Then, the allocation information (randomization results) was concealed in opaque and sealed envelopes by the statistician. The observer (A.M.) who performed all the measurements was blinded to the group assignment. The doctor who treated the patients were blinded to the group assignment. The study was blinded about the statistical analysis: blinding was obtained by eliminating from the elaboration file every reference to patient group assignment**.**

**Figure 4:**
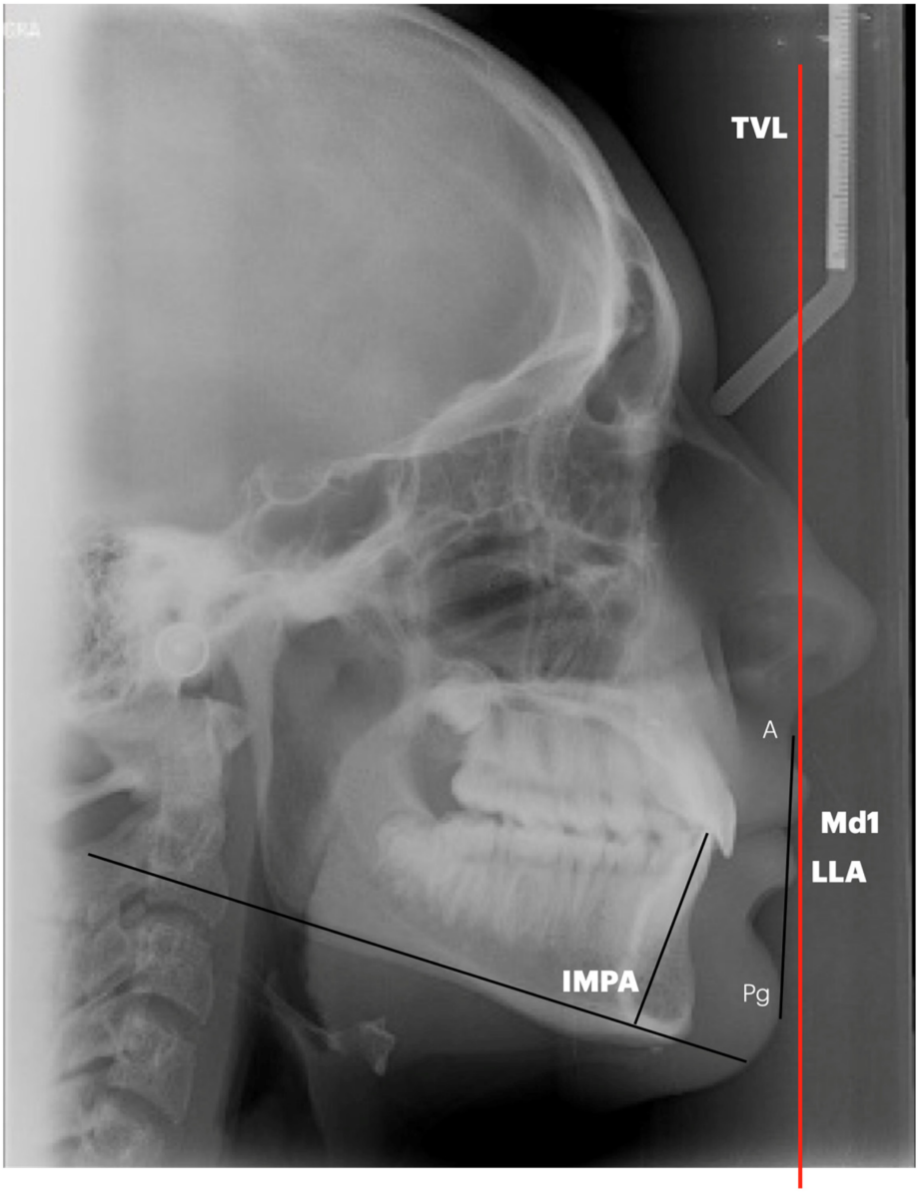
Cephalometric parameters evaluated.

**Figure 5:**
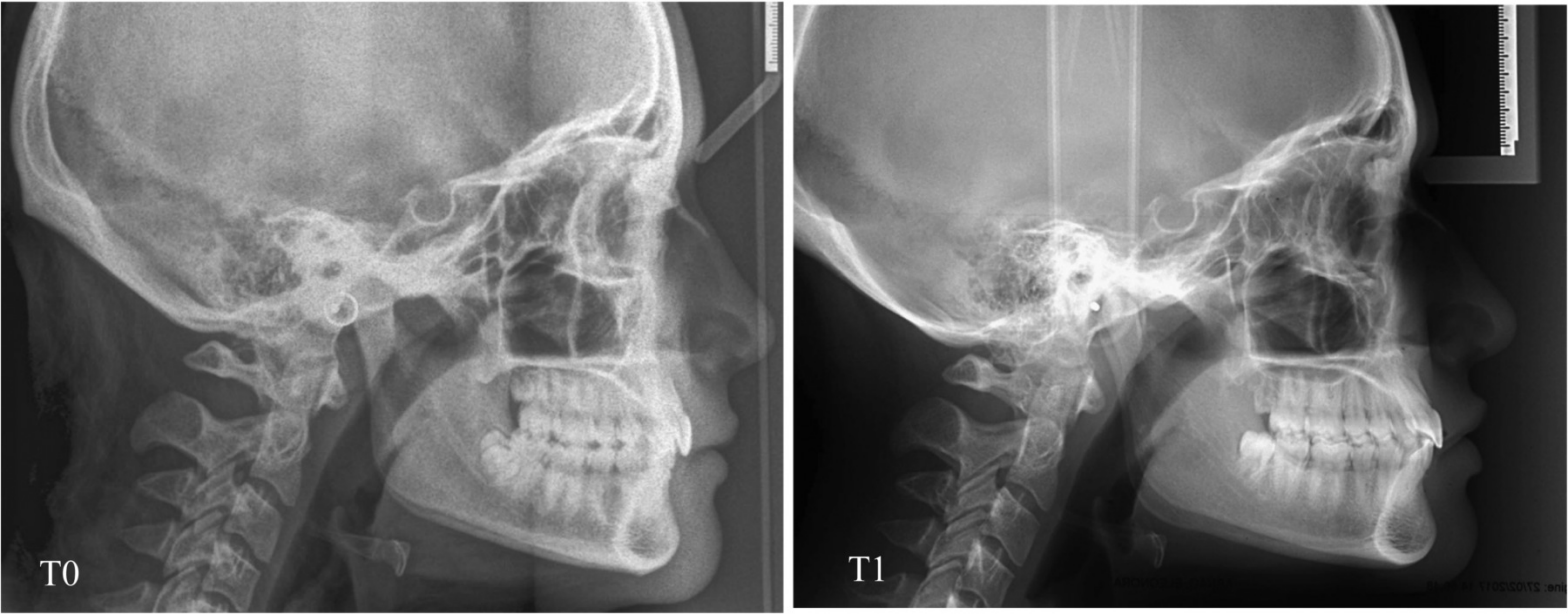
Lateral cephalometric radiography at time T0 and T1 of a patient in the MB.

**Figure 6:**
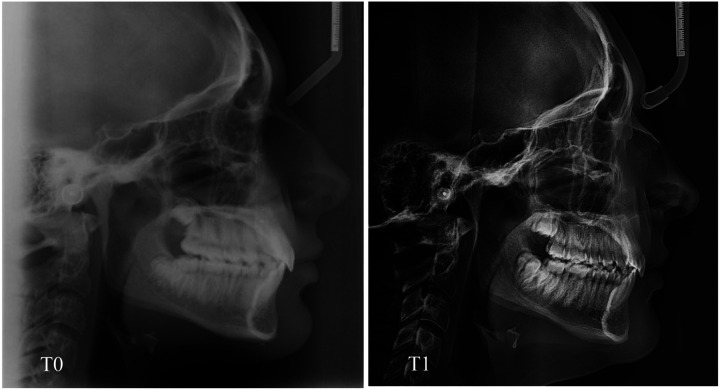
Lateral cephalometric radiography at time T0 and T1 of a patient in the AG.

### Data measurements

Radiographs were manually traced by the same expert operator (A.M.) blinded about the study. A total of four cephalometric parameters (1 angular, 3 linear) were measured and recorded for each cephalogram (Figure 4):

- **IMPA**, angle formed by the intersection of the axis of the lower incisor with the Tweed mandibular plane
- **L1/A-Pg**, position of lower incisor to A-Pogonion line
- **Md1-TVL**, position of lower incisor to Arnett True Vertical line
- **LLA-TVL**, position of lower lip to Arnett True Vertical line.

### Statistical analysis

To determine reproducibility of the method, the same cephalometric analysis was performed on all radiographs for all patients. The same operator (A.M.) re-traced again the radiohraphy ten days after the first time. A paired *t*-Test was used to compare the two measurements (systematic error). The magnitude of the random error was calculated using the reliability coefficient.

Descriptive statistics were calculated for all measurements in each group. Exploratory statistics revealed that all variables were normally distributed (Kolmogorov–Smirnov test) with equality of variances (Levene's test).

Student *t*-Test was used to compare the means of the quantitative variables associated with the effect of the device over time T1-T0. Statistics, correlations and finally tests for paired samples were developed. In the presence of normally distributed data, descriptive statistics were calculated for each measurement in each group and significant between-group differences were tested with the independent sample Student's *t* test. The level of significance was set at 5%.

## Results

No systematic error was found between the repeated digital measurements. It was reduced by precise definitions of points in the presence of a previously trained examiner. The intra-observer reliability ranged between 0.32° and 1.22° for cephalometric angular measurements and between 0.51 mm and 1.01 mm for linear measurements. There was no systematic error for any measurement Student's *t*-test: *p* > 0.01). ICC ranged between 0.770 to 0.999.

As reported in Table 1, the analysis of the starting forms showed no statistically significant differences at T0 between the groups for all measurements.

**Table 1:** Starting forms for linear and angular measurements between fixed appliance sample (**MBG**) and clear aligner sample (**AG**).

Variables	MBG		AG		Statistical analysis results between MGB and AG		
	(*n* = 20; 9M, 11F)		(*n* = 20; 12M, 8F)				
	Mean	SD	Mean	SD	Diff	SD	*P*-value
IMPA	90.3°	1.2	94.2°	1.1	−3.9	1	NS
L1/A-Pg	1.9 mm	1.3	1.8 mm	0.9	0.1	0.7	NS
Md1-TVL	−15.7 mm	2.3	−16.8 mm	2.1	−1.1	0.9	NS
LLA-TVL	−2.7 mm	0.8	−2.5 mm	0.6	−0.2	0.5	NS

NS, not significant; SD, standard deviation; Diff., differences.

In the MBG, at the end of therapy (T1-T0 = 18 months), the results showed no statistically significant changes in any of the evaluated parameters (*p* > 0.05), as shown in Table 2.

**Table 2:** Descriptive statistics and statistical comparisons of the T0-T1 changes in **MBG**.

MBG	T0	T1	T1-T0	*P*-value
	(Mean)	(Mean)	(Diff)	
IMPA	90.3°	94.5°	4.2°	0.15
L1/A-Pg	1.9 mm	2.4 mm	0.5 mm	0.32
Md1-TVL	−15.7 mm	−15.3 mm	−0.4 mm	0.71
LLA-TVL	−2.7 mm	−3-0 mm	-0.3 mm	0.88

Diff, differences.

However, a more important, though non-significant, increase in the IMPA angle value can be observed in the MBG compared with the AG (difference = 3.5°, *p* = 0.27).

As for the AG, the data calculated at the two different times (T0, T1) were compared and the following results were observed (Table 3): no changes in the average of the IMPA angle (*p* = 0.39), L1/A-Pg (*p* = 0.28), Md1-TVL (*p* = 0.86), LLA-TVLL (*p* = 0.93) values. At the end of the therapy (T1-T0 = 18 months) the analysis of the results showed no statistically significant changes in all evaluated parameters (*p* > 0.05).

**Table 3:** Descriptive statistics and statistical comparisons of the T1-T0 changes in aligner (AG) group.

AG	T0	T1	T1-T0	*P*-value
	(Mean)	(Mean)	(Diff)	
IMPA	94.2°	94.9°	0.7°	0.39
L1/A-Pg	1.8mm	2.6 mm	0.8 mm	0.28
Md1-TVL	−16.8 mm	−16.6 mm	−0.2 mm	0.86
LLA-TVL	−2.5 mm	−2.6 mm	−0.1 mm	0.93

Diff, differences.

The comparison of T1 changes in MBG and AG showed no significant differences in all parameters evaluated (Table 4).

**Table 4:** Descriptive statistics and statistical comparisons of the T1 changes in **MBG** and **AG.**

Variables	MBG	AG	Diff	*P*-value
IMPA	94.5°	94.9°	0.4	NS
L1/A-Pg	2.4 mm	2.6 mm	0.2	NS
Md1-TVL	−15.3 mm	−16.6 mm	−1.1	NS
LLA-TVL	−3-0 mm	−2.6 mm	−0.2	NS

Diff, differences; NS, not significant.

## Discussion

The purpose of this randomized clinical trial was to analyze changes in lower incisors' inclination in a group of adolescent patients with Class II malocclusion treated with two different treatment protocols: Class II elastics and clear aligners compared with a group treated with Class II elastics and fixed appliance. The results of the present study showed that both treatments provide good control of the inclination of the lower incisors (Figures 5, 6) which is a key factor in this type of treatment, considering that in Class II dento-alveolar corrections with intermaxillary elastics, the proclination of the lower incisors is often an undesirable effect (15).

Although the use of aligners continues to grow quickly, there is still little evidence in the literature evaluating the effects of these devices in treatment with Class II elastics (17). Nevertheless, the results of the present study on changes in lower incisor inclination after Class II elastics are similar to those reported in the literature (15, 17). No statistically significant differences were found for any of the parameter examined, so both methods presented good control of lower incisor position with respect to both chin symphysis and TVL. The position of the upper and lower incisors is one of the main aspects of orthodontic treatment, as it is an important determinant for the patient's facial appearance (18). In fact, the position and esthetics of the lips can be influenced by that of the incisors, and this could be considered when establishing the treatment goals. Furthermore, when determining the final position of the incisors, an excessively proclined incisor should be avoided because of the risk of moving the teeth out of the alveolar envelope and developing a bone dehiscence, thus creating the risk of gingival recession (19). Expanding the arches and proclining teeth is a viable alternative to extractions for space recovery. Many authors, however, observed that the position of the incisors into the alveolar bone can influence the gingival attachment and long-term stability (20).

Dianiskova et al. in 2022 showed that the use of clear aligners provides better control of the lower incisors than fixed braces. Furthermore, the same authors suggested that the two treatments achieved similar results in patients with buccal tipping of the lower incisors (15).

Unlike aligners, braces exert coronal and buccal force relative to the center of resistance of the teeth (17, 21), this may cause tipping and proclination during alignment. Several methods can be used to control the position of the lower incisors with fixed appliances, such as IPR, placing bend backs in arch wires, performing lace backs or using negative torque prescription brackets for lower incisors. Clear aligners can align the teeth, moving one or more teeth and this gradual, segmented movement could minimize tooth proclination. So, it could be hypothesized that clear aligners are suitable for patients with thin gingival biotypes to limit the risk of gingival recession (22).

Considering the results of the present study, the two types of treatment achieved similar results; in fact, there were no differences when comparing the parameters examined between the two studied groups: clear aligners and fixed appliance therapy allow to control the inclination of the lower incisors during the treatment. It is up to the clinician to choose the right type of treatment based on the patient's characteristics. Importantly, the AG presented a good control of IMPA angle as the change in IMPA angle is less in the AG (0.7°) than in the MBG (4.2°) with a difference of 3.5°, even if this difference not statistically significant. Thus further studies are needed to confirm this observation. One of the side effects in Class II elastics treatment with fixed therapy is precisely proclination, as reported in several studies. About clear aligner al Class II elastics, Liu X et al. in 2022 demonstrated that under the conditions of Class II elastics, the mandibular anterior teeth experienced undesirable labial movement (23). In some cases, this proclination is considered a desired movement, for example, to correct Class II malocclusion with a deep bite and retroclined lower incisors; however, in some cases, this proclination is undesirable. For example, in hyperdivergent patients there is often a Class II malocclusion due to clockwise rotation of the mandible, which is associated with crowding and tilted lower incisors. In this case, proclination of the lower incisors may be considered an undesirable movement that could harm the patient and create recession of the lower incisors.

This study has an important clinical relevance ad it showed that the use of clear aligners with intermaxillary elastics is an important treatment option for certain types of patients when the position of the lower incisors needs to be maintained. Probably, the increased control is associated with the aligner's rigidity in keeping the entire arch locked, or with a better distribution of the forces produced by the elastics on the aligner than with brackets (24, 25).

Thus, it can be assumed that due to its structure, the clear aligners avoid the proclination of the lower incisors due to Class II elastics.

The good control of the inclination of the lower teeth offered by the clear aligners could be associated with the aligner structure containing and fully covering the clinical crown of the teeth. In addition, digital planning with clear aligners allows monitoring of tooth movement during treatment (26). Considering that lower incisor proclination is one of the main problems with Class II rubber band treatments and fixed multibrackets therapy, aligners seem to offer an advantage when lower incisor inclination is undesirable (15).

### Limitations

The current trial had some limitations: it was not possible to fully control patients' compliance in wearing aligners in the aligner group and wearing elastics in the MBG and AG. However, the final Class I occlusion confirmed that the patients' clinical conditions were in accordance with the expected results at the end of both types of therapy. Therefore, further studies are needed to investigate this topic to provide clear directions for treatment with Class II elastics and analyze the stability of long-term results obtained in both MBG and AG.

## Conclusion

The present data suggest that Class II elastics combined with clear aligners or fixed appliance produced a similar control in the proclination of the lower incisors. There were no statistically significant differences between both MBG and AG.

The results of the present clinical trial can be generalized for patient groups presenting similar mean age, inclusion/exclusion criteria, and type of treatment protocol.

## Data Availability

The original contributions presented in the study are included in the article/Supplementary Material, further inquiries can be directed to the corresponding author.
